# Supplementary material for: A large-scale genome and transcriptome sequencing analysis reveals the mutation landscapes induced by high-activity adenine base editors in plants
Source: Genome Biol. 2022 Feb 9;23:51. doi: 10.1186/s13059-022-02618-w (PMC8826654; doi:10.1186/s13059-022-02618-w)
Supplement: Supplementary file 2 — Additional file 2: Supplementary figures. Fig. S1. Sanger sequencing chromatograms of on-target mutations in plants harboring rBE46b and rBE49b. Fig. S2. Sanger sequencing chromatograms of on-target mutations in plants harboring rBE50 and rBE53. Fig. S3. IGV browser views showing the on-target mutations for 36 plants harboring ABEs. Fig. S4. Analysis of SNVs and indels identified by whole-genome sequencing. Fig. S5. Analysis of the remaining background homozygous DNA mutations. Fig. S6. Characterization of ABE-induced genomic mutations. Fig. S7. Distribution of six types of SNVs. Fig. S8. Distribution of SNVs at given regions of the genome. Fig. S9. Chromosomal distribution of SNVs. Fig. S10. On-target and off-target mutations in plants from the same calli. Fig. S11. Off-target SNVs in plants with incomplete T-DNA insertions. Fig. S12. Distribution of SNVs with different copy numbers of T-DNA insertions. Fig. S13. Transcriptome-wide distribution of ABE-induced off-target mutations. Fig. S14. Heatmap demonstrating A>G mutations in transcriptomes with more than 5 A>G SNVs detected. Fig. S15. The 5′ and 3′ flanking A>G mutations in transcriptomes with ABEs containing A>G RNA SNVs and in transcriptomes with SpCas9 only lacking A>G RNA SNVs. Fig. S16. The 5′ and 3′ flanking A>G mutations in transcriptomes with ABEs but without A>G RNA SNVs. Fig. S17. IGV genome browser views showing the off-target RNA mutations. Fig. S18. IGV genome browser views showing A>G mutations with flanking A>G SNVs in genome sequencing data. Fig. S19. Sanger sequencing chromatograms of off-target A>G DNA mutations. Fig. S20. Sanger sequencing chromatograms of off-target A>G RNA mutations. [file 13059_2022_2618_MOESM2_ESM.docx]

**
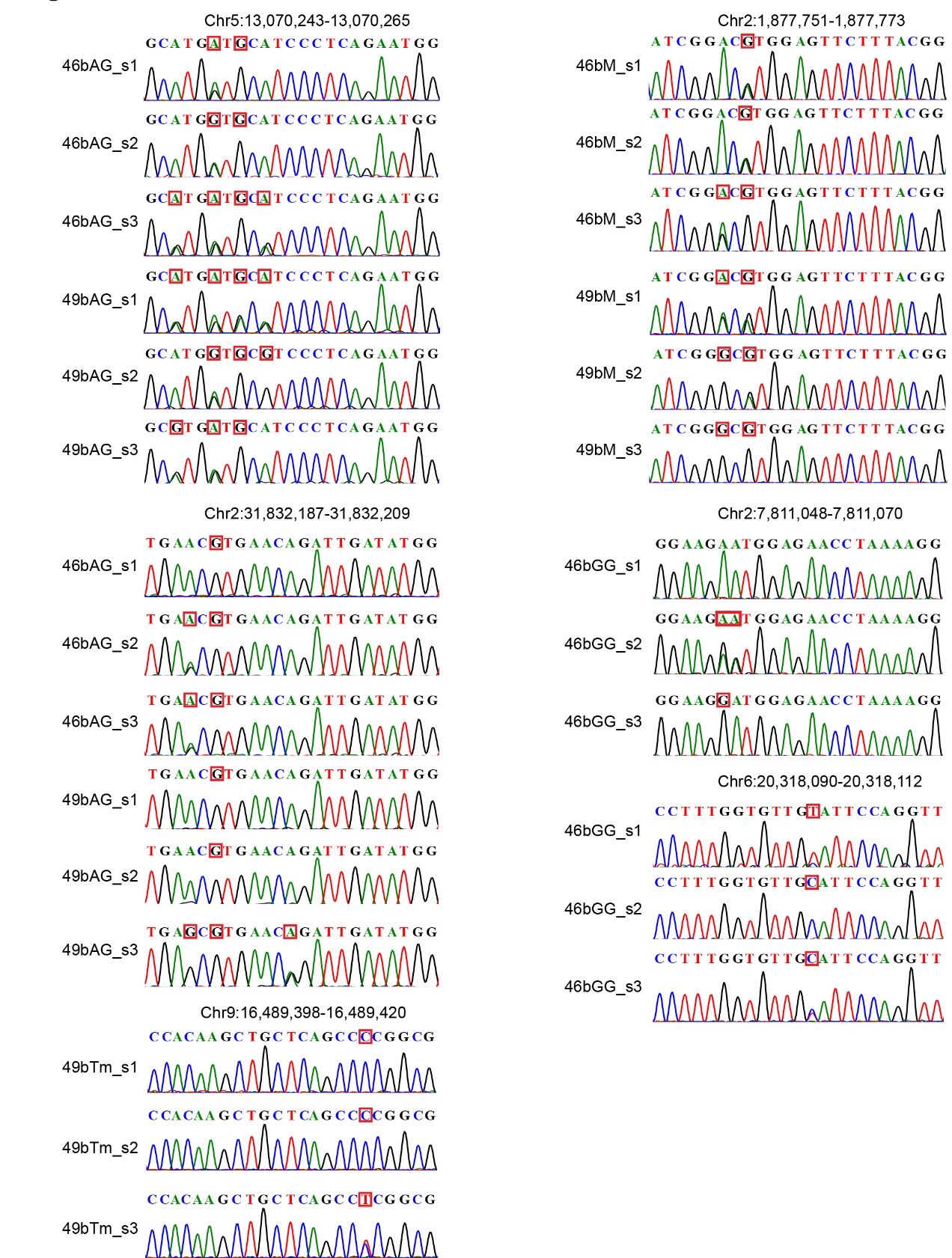
**

**Figure S1. Sanger sequencing chromatograms of on-target mutations in plants harboring rBE46b and rBE49b.**

**
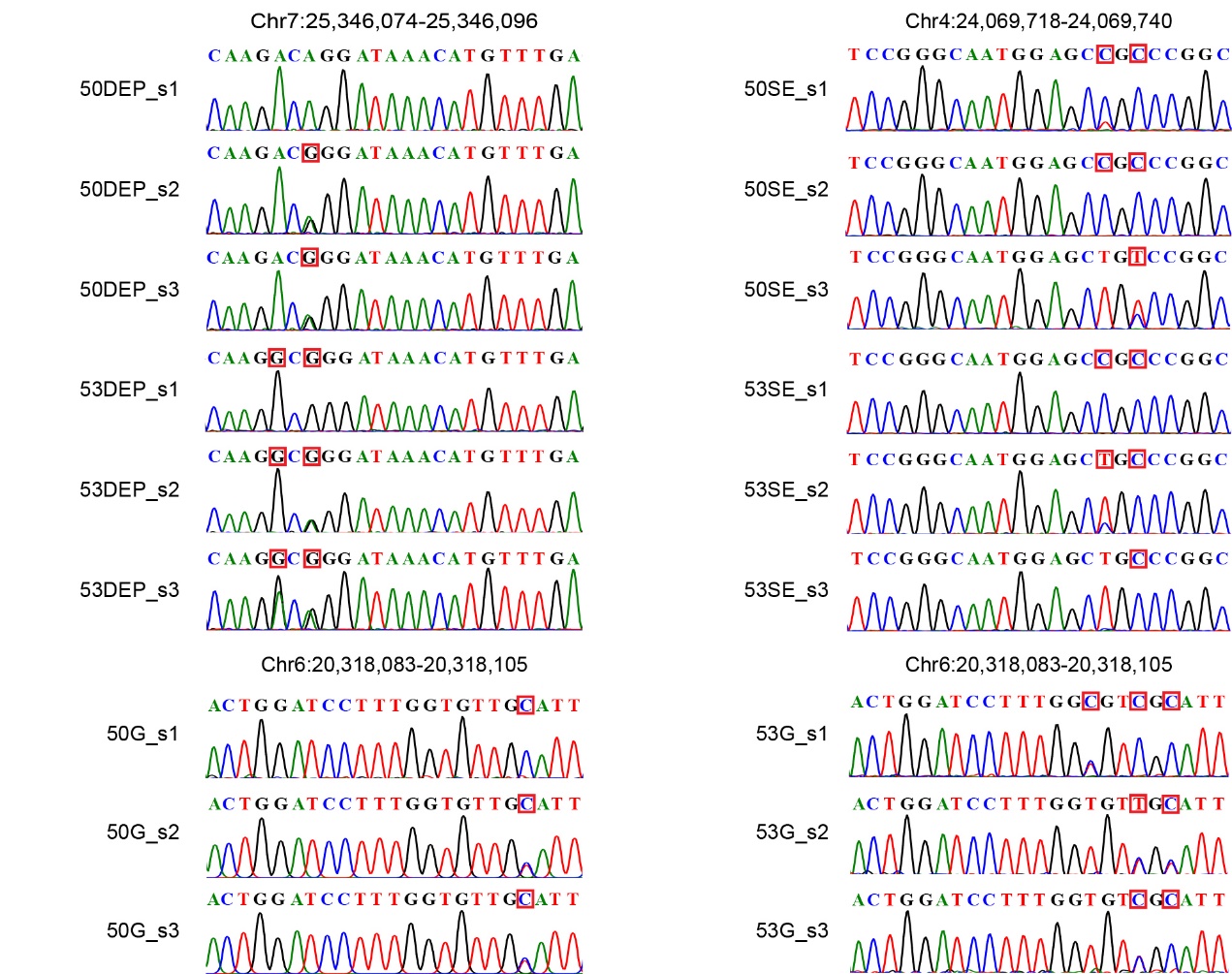
**

**Figure S2. Sanger sequencing chromatograms of on-target mutations in plants harboring rBE50 and rBE53.**

**
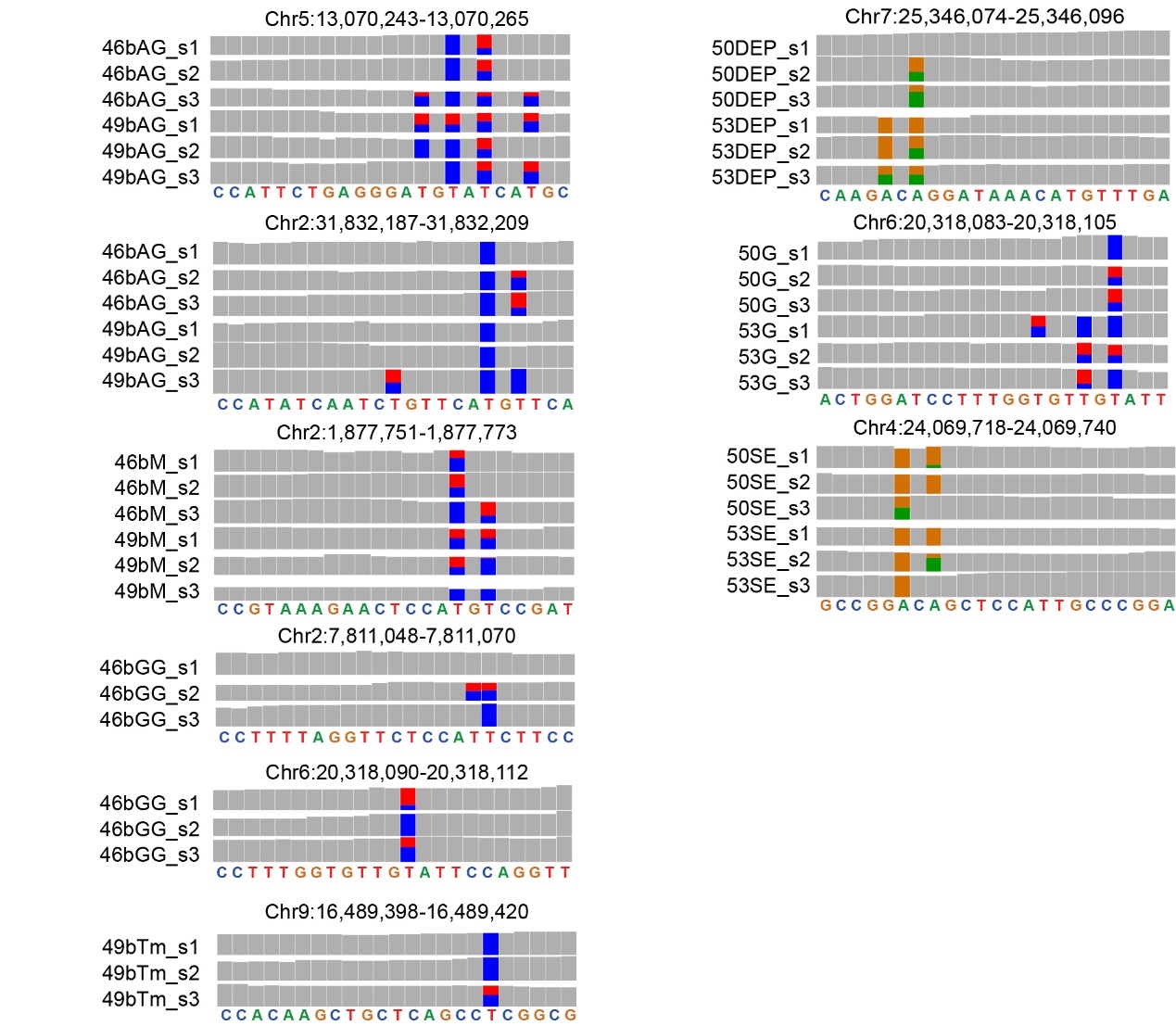
**

**Figure S3. IGV browser views showing the on-target mutations for 36 plants harboring ABEs.** In IGV genome browser views, the grey bar represents a sequenced nucleotide that is the same as the reference genome, while bars in other colors represent sequenced nucleotides that are partially or totally different from the reference genome: red represents nucleotide T, green represents nucleotide A, orange represents nucleotide G, and blue represents nucleotide C. The height of each color bar represents the relative composition of each nucleotide.


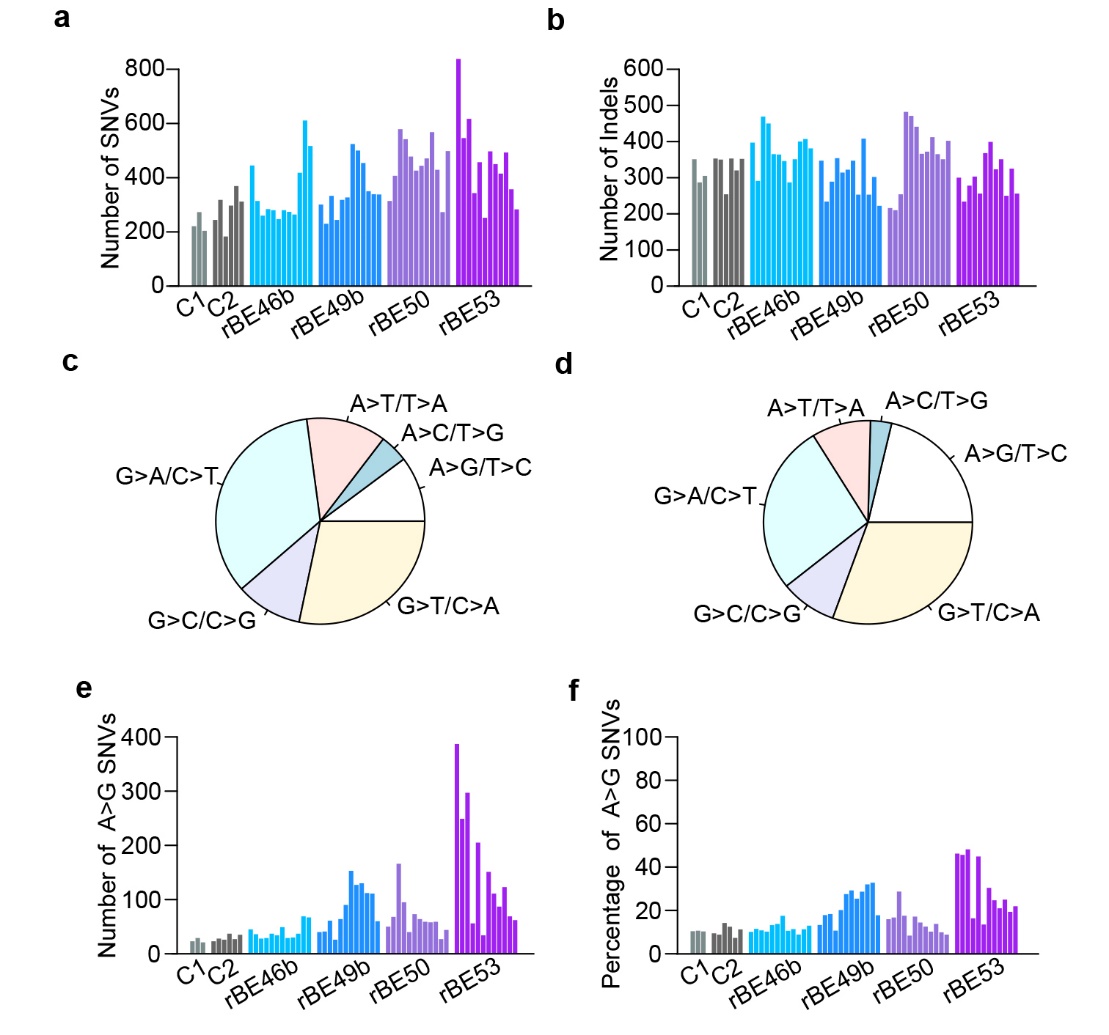


**Figure S4. Analysis of SNVs and indels identified by whole-genome sequencing.** **a-b** Number of SNVs and indels induced by tissue culture (C1), *Agrobacterium* infection (C2), and the presence of rBE46b (SpCas9n-TadA8e), rBE49b (SpCas9n-TadA9), rBE50 (SpCas9n-NG-TadA8e), and rBE53 (SpCas9n-NG-TadA9). **c** Pie chart showing the distribution of six types of SNVs induced by tissue culture and *Agrobacterium* infection. **d** Pie chart showing the distribution of six types of SNVs induced by ABEs. **e** Number of A>G SNVs in each of the sequenced plants. **f** Percentage of A>G SNVs in each of the sequenced plants. **a, b, e and f** Plants in C1 are in the following order: C1_s1, C1_s2, and C1_s3. Plants in C2 are in the following order: C2_s1, C2_s2, C2_s3, C2_s4, C2_s5, and C2_s6. Plants in rBE46b are in the following order: 46bAG_s1, 46bAG_s2, 46bAG_s3, 46bGG_s1, 46bGG_s2, 46bGG_s3, 46bM_s1, 46bM_s2, 46bM_s3, 46bg_s1, 46bg_s2, and 46bg_s3. Plants in rBE49b are in the following order: 49bAG_s1, 49bAG_s2, 49bAG_s3, 49bM_s1, 49bM_s2, 49bM_s3, 49bTm_s1, 49bTm_s2, 49bTm_s3, 49bg_s1, 49bg_s2, and 49bg_s3. Plants in rBE50 are in the following order: 50DEP_s1, 50DEP_s2, 50DEP_s3, 50G_s1, 50G_s2, 50G_s3, 50SE_s1, 50SE_s2, 50SE_s3, 50bg_s1, 50bg_s2, and 50bg_s3. Plants in rBE53 are in the following order: 53DEP_s1, 53DEP_s2, 53DEP_s3, 53G_s1, 53G_s2, 53G_s3, 53SE_s1, 53SE_s2, 53SE_s3, 53bg_s1, 53bg_s2, and 53bg_s3.


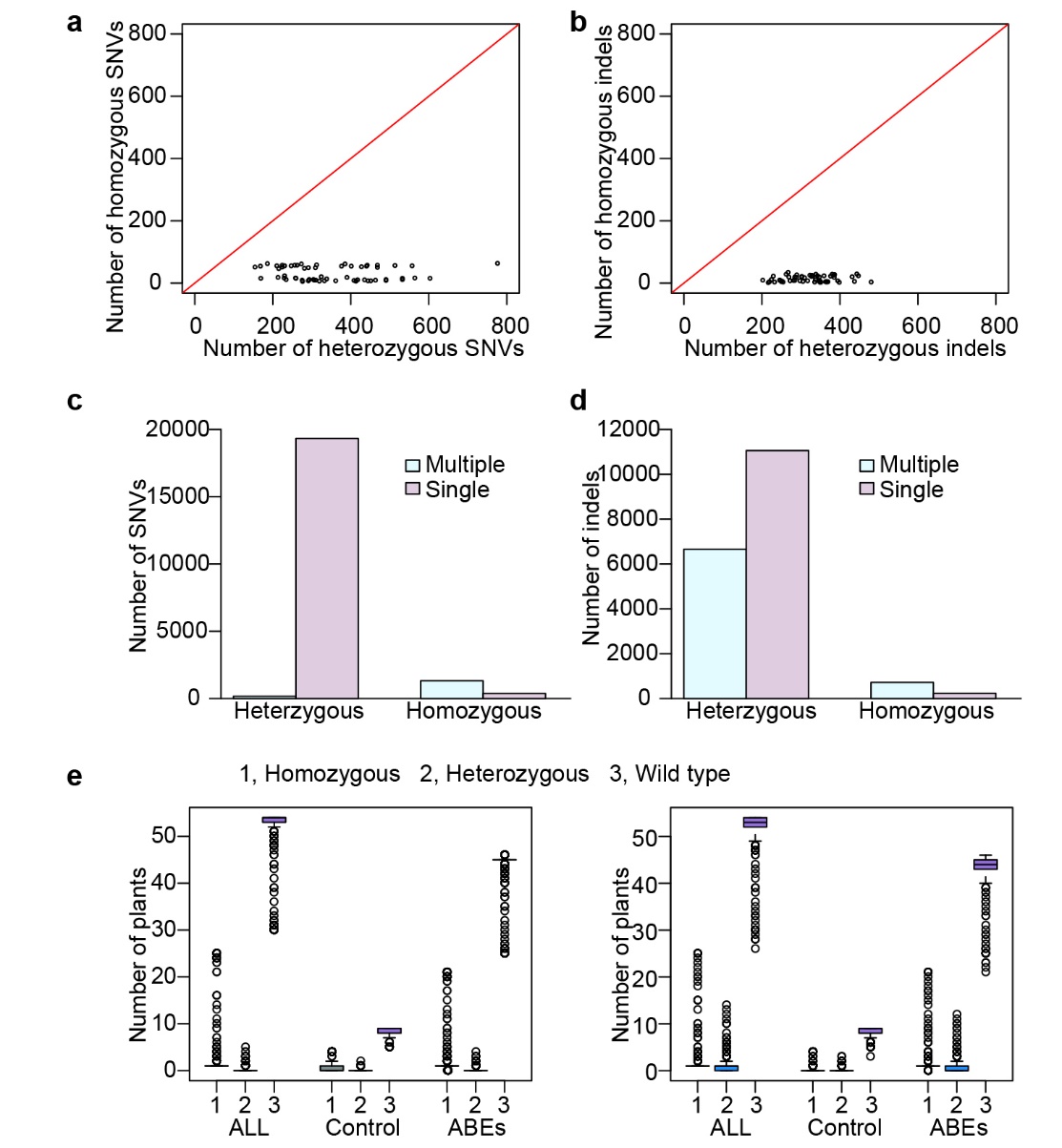


**Figure S5. Analysis of the remaining background homozygous DNA mutations. a** Scatterplot showing the distribution of homozygous and heterozygous SNVs in all sequenced plants. **b** Scatterplot showing the distribution of homozygous and heterozygous indels in all sequenced plants. **c-d** Bar graphs showing the occurrence of SNVs and indels in all sequenced plants. Light-blue bars represent the number of SNVs in more than one plant (Multiple). Mauve bars represent the number of SNVs in one single plant (Single). **e** Boxplots showing the number of plants with homozygous, heterozygous, and wild-type SNVs or indels at the homozygous mutation loci.

**
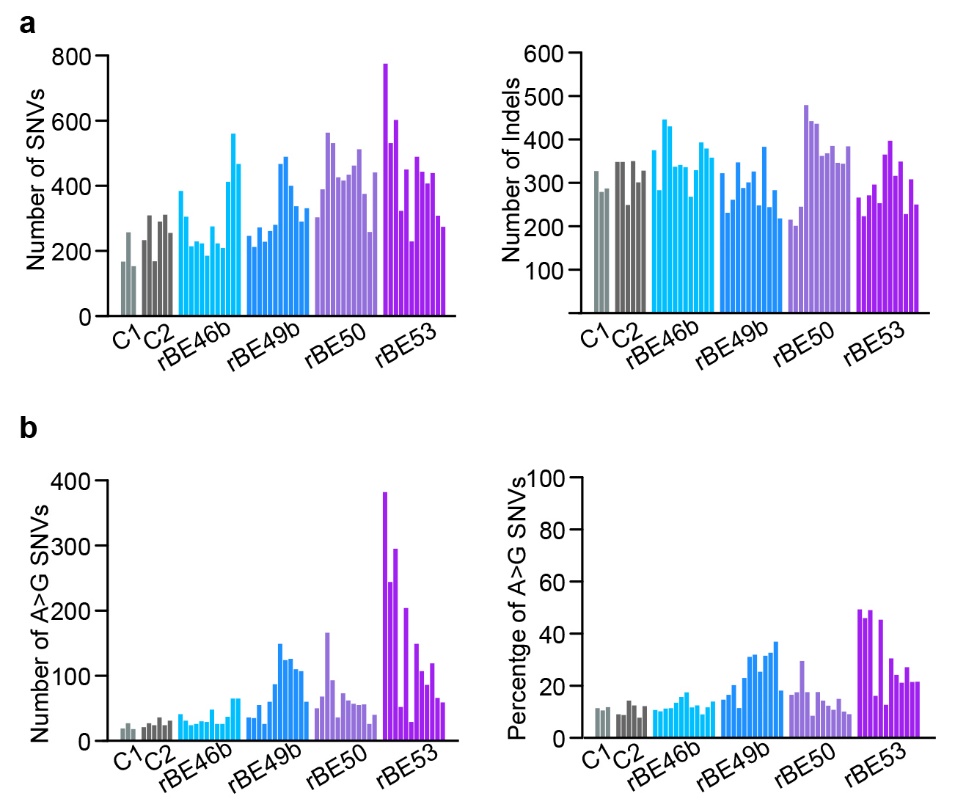
**

**Figure S6. Characterization of ABE-induced genomic mutations. a** Number of SNVs and indels induced by tissue culture (C1), *Agrobacterium* infection (C2), and the presence of rBE46b, rBE49b, rBE50, and rBE53. **b** Bar graphs showing the number and percentage of A>G SNVs identified in different groups of plants. **a** and **b** Plants are in the same order as in Additional File 1: Figure S4.

**
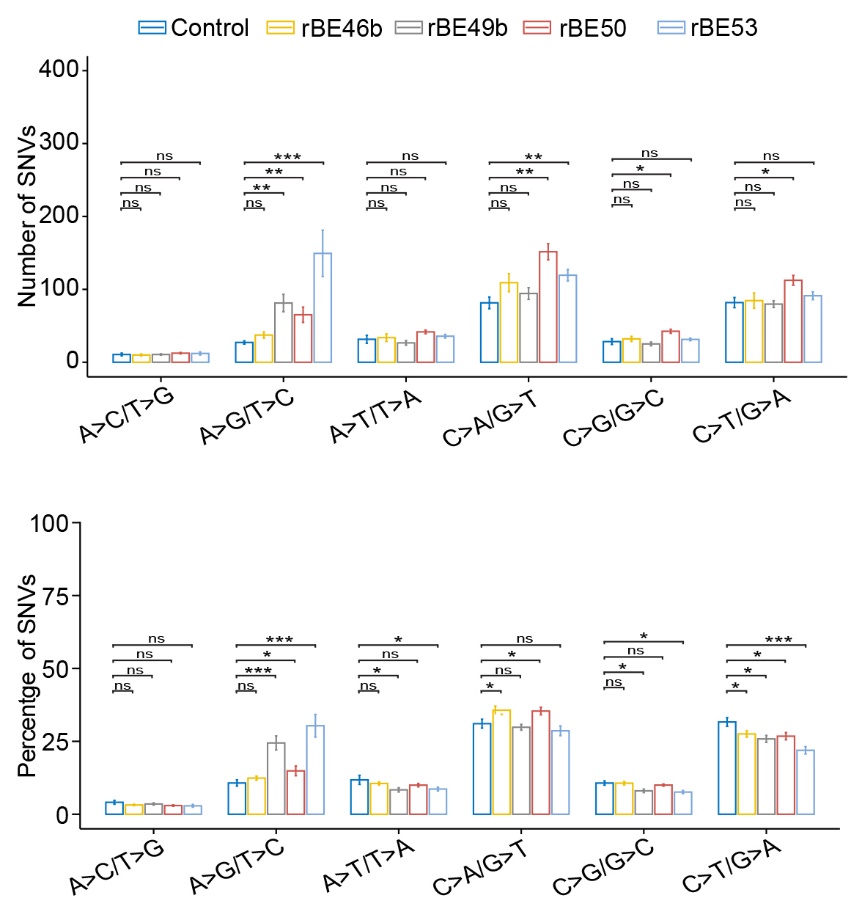
**

**Figure S7. Distribution of six types of SNVs.** Number and percentage of six types of SNVs in plants that have undergone *Agrobacterium* infection (Control) and harbor rBE46b, rBE49b, rBE50, and rBE53. Each bar represents the mean value; each error bar represents the standard error. (ns) denotes *p*-value > 0.1, (*) denotes *p*-value < 0.1, (**) denotes *p*-value < 0.01, and (***) denotes *p*-value < 0.001 (one-tailed Wilcoxon test).

**
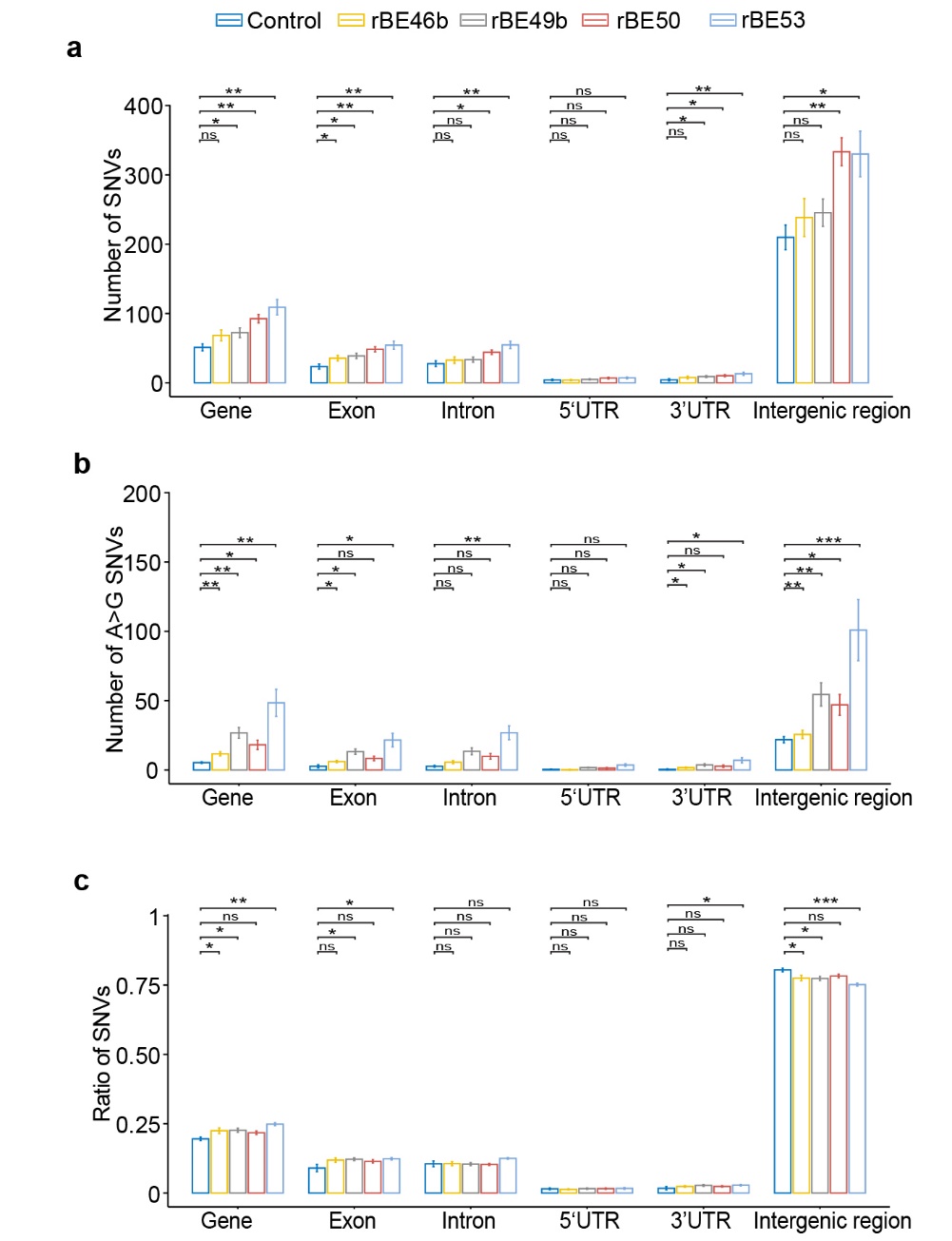
**

**Figure S8. Distribution of SNVs at given regions of the genome. a** Number of SNVs at given regions in control groups and four types of ABEs. **b-c** Number and percentage of A>G SNVs at given regions in control groups and four types of ABEs. Each bar represents the mean value; each error bar represents the standard error. (ns) denotes *p*-value > 0.1, (*) denotes *p*-value < 0.1, (**) denotes *p*-value < 0.01, and (***) denotes *p*-value < 0.001 (one-tailed Wilcoxon test).

**
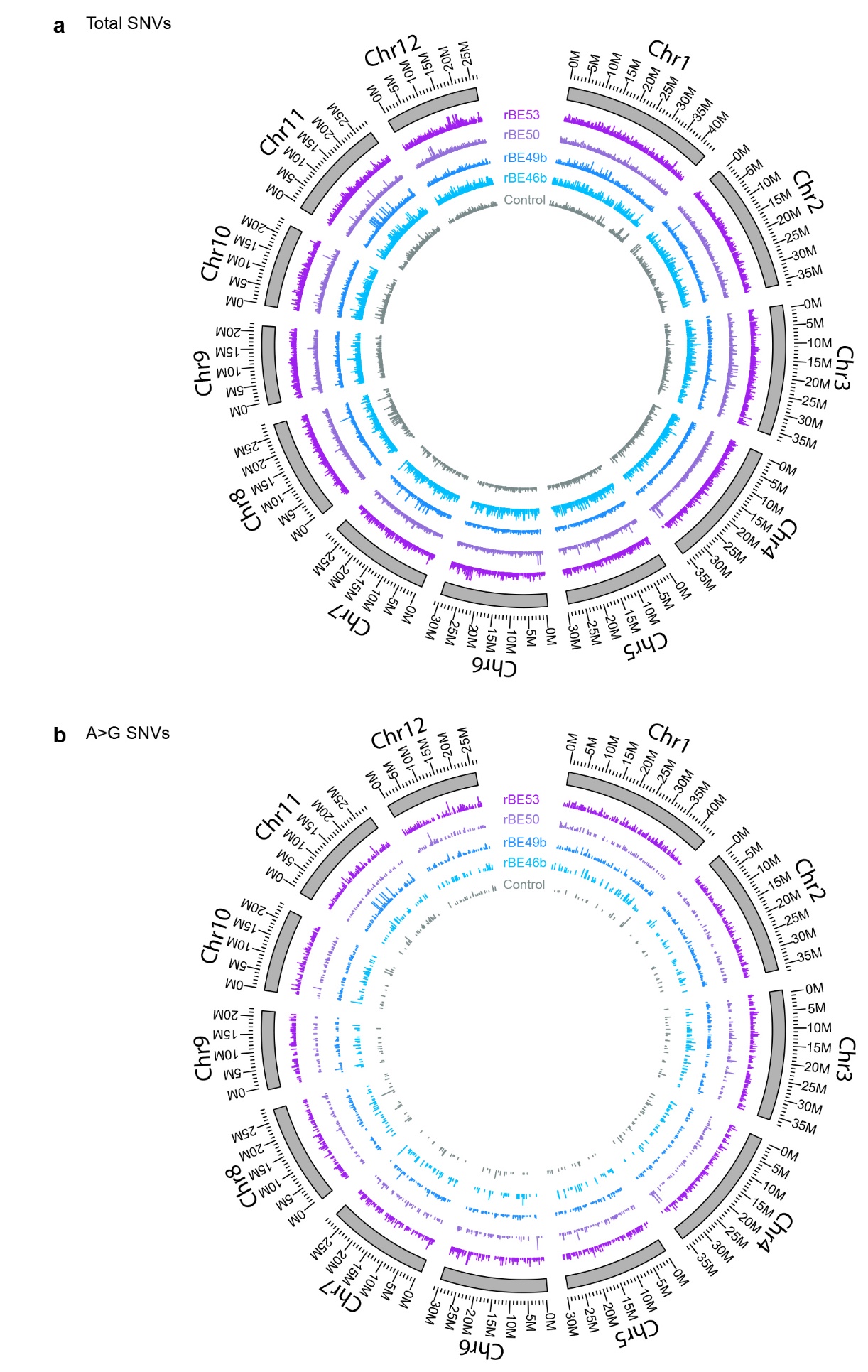
**

**Figure S9. Chromosomal distribution of SNVs. a-b** Chromosomal distribution of SNVs and A>G SNVs in control groups and in plants harboring four types of ABEs. The height was calculated based on the density in fixed 5,000 bp windows.

**
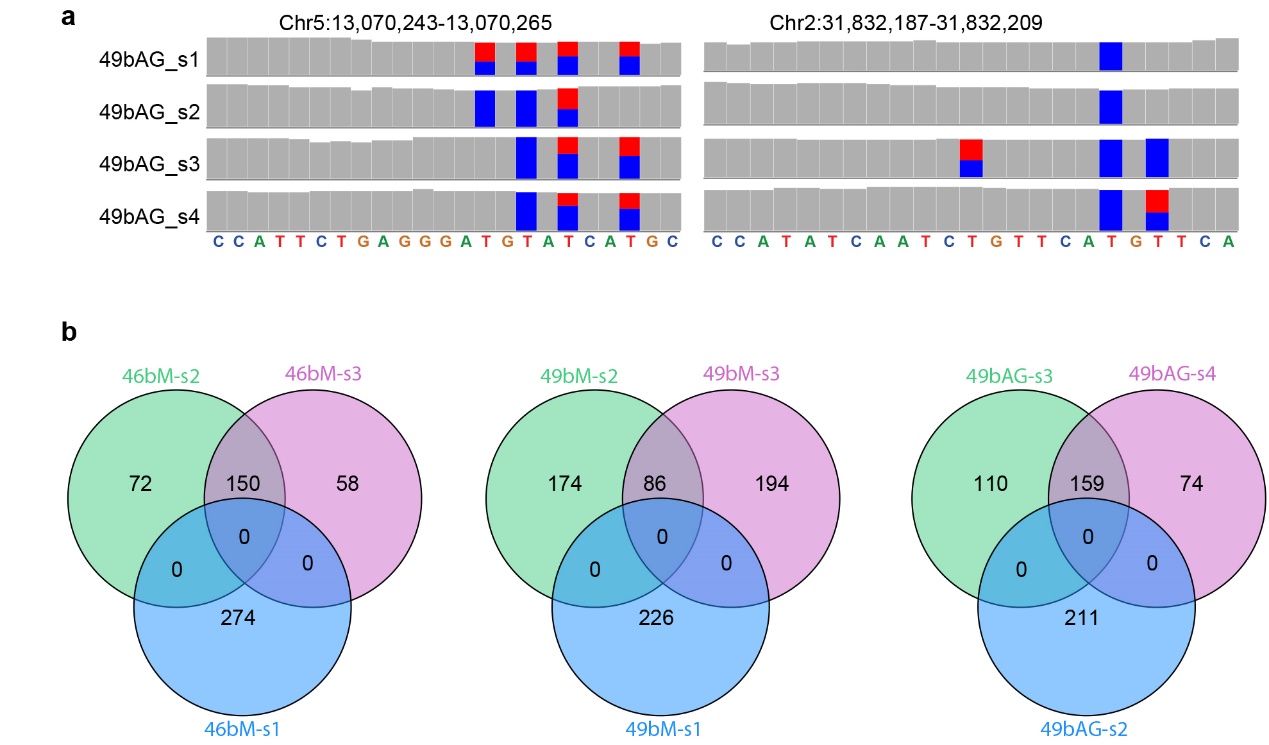
**

**Figure S10. On-target and off-target mutations in plants from the same calli. a** IGV browser views showing the on-target mutations in plants carrying the 49bAG construct. **b** Venn diagrams showing the overlapping off-target SNVs among three plants with the same construct from the same calli or different calli. In IGV genome browser views, the grey bar represents a sequenced nucleotide that is the same as the reference genome, while bars in other colors represent sequenced nucleotides that are partially or totally different from the reference genome: red represents nucleotide T, blue represents nucleotide C. The height of each color bar represents the relative composition of each nucleotide.


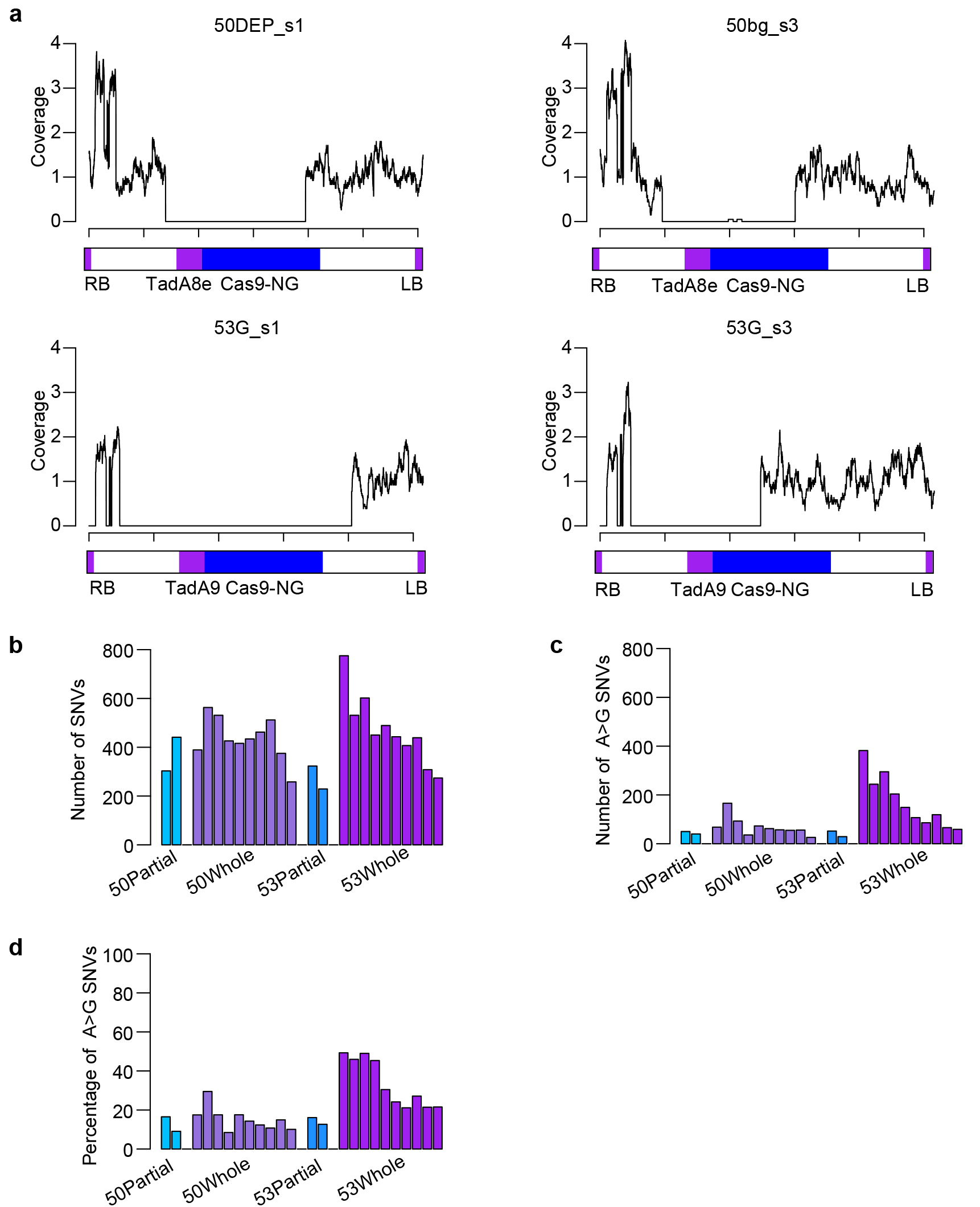


**Figure S11. Off-target SNVs in plants with incomplete T-DNA insertions. a** The T-DNA coverage between the right border (RB) and left border (LB) in plants with an incomplete T-DNA insertion. **b-d** Plants with rBE50 and rBE53 were divided into two groups. Partial represents plants with incomplete T-DNA insertion, while whole represents plants with complete T-DNA insertion. The number of SNVs and A>G SNVs, and the percentage of A>G SNVs were determined from each of these plants. Plants in 50Partial are in the following order: 50DEP_s1 and 50bg_s3. Plants in 50Whole are in the following order: 50DEP_s2, 50DEP_s3, 50G_s1, 50G_s2, 50G_s3, 50SE_s1, 50SE_s2, 50SE_s3, 50bg_s1, and 50bg_s2. Plants in 53Partial are in the following order: 53G_s1 and 53G_s3. Plants in 53Whole are in the following order: 53DEP_s1, 53DEP_s2, 53DEP_s3, 53G_s2, 53SE_s1, 53SE_s2, 53SE_s3, 53bg_s1, 53bg_s2, and 53bg_s3.

**
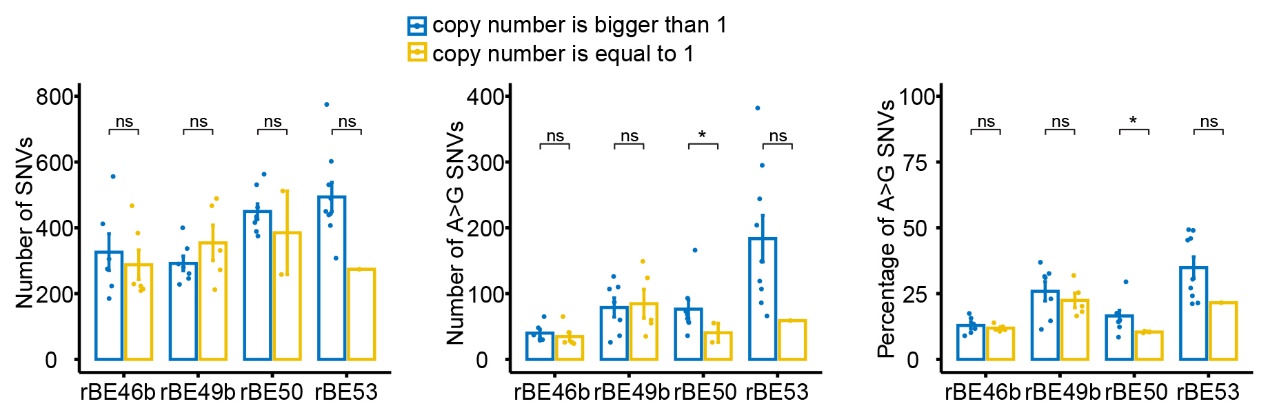
**

**Figure S12. Distribution of SNVs with different copy numbers of T-DNA insertions.** Plants transformed with rBE46b, rBE49b, rBE50 and rBE53 were divided to two groups. One group contains plants with one copy of T-DNA insertions and the other groups contains plants with multiple copies of T-DNA insertions. The number of total SNVs, the number of A>G SNVs and percentage of A>G SNVs were compared between these two groups. (ns) denotes *p*-value > 0.1, (*) denotes *p*-value < 0.1 (one-tailed Wilcoxon test).

**
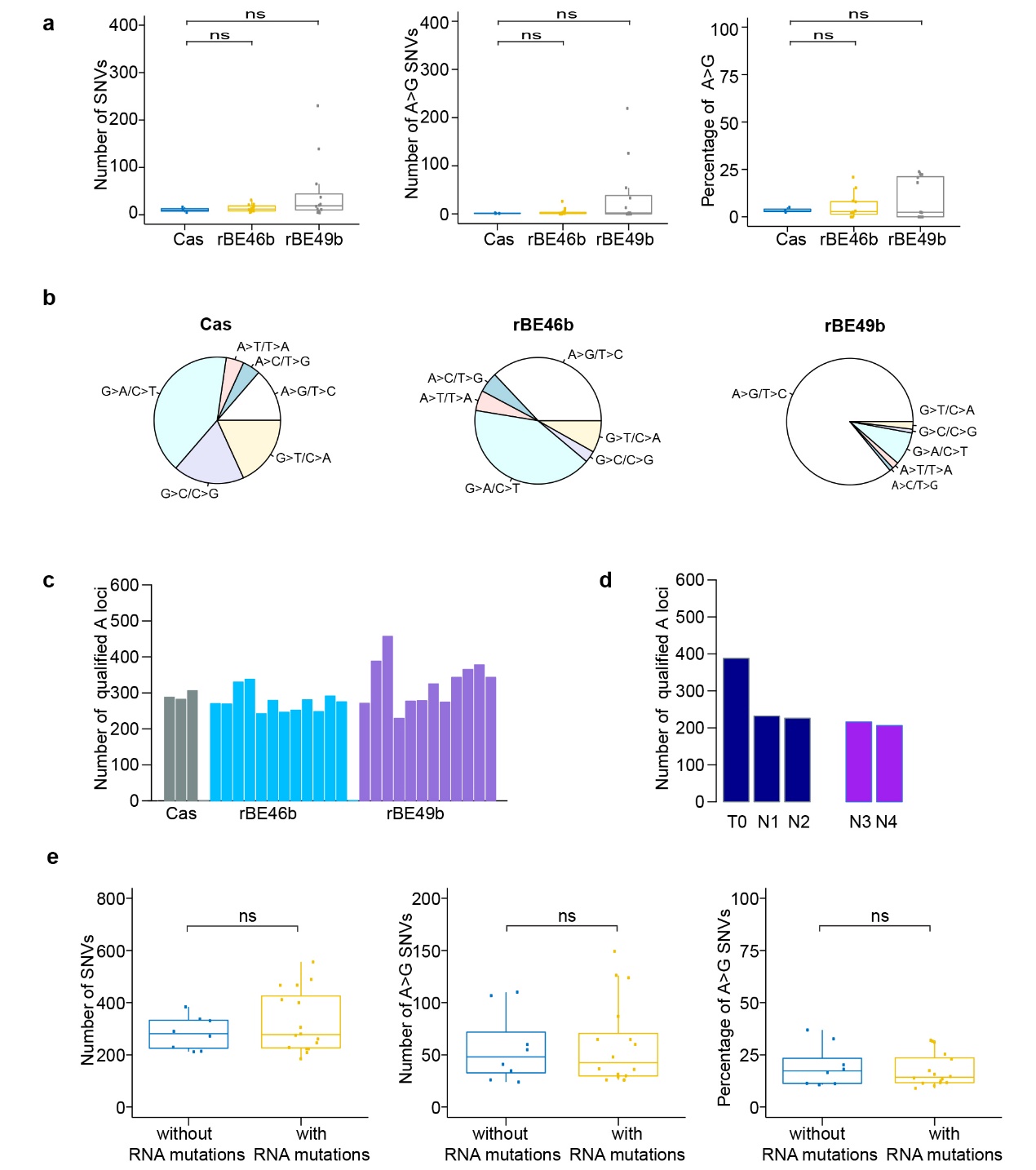
**

**Figure S13. Transcriptome-wide distribution of ABE-induced off-target mutations. a** Number of SNVs and A>G SNVs, and percentage of A>G SNVs in plants harboring SpCas9 only, rBE46b, and rBE49b. Each dot represents the number of SNVs and A>G SNVs, and the percentage of A>G SNVs from an individual plant. **b** Pie charts showing the distribution of six types of SNVs in plants with SpCas9, rBE46b, and rBE49b separately. **c** Number of nucleotide A/T loci with more than 10 reads to be qualified for calculating the frequency of A>G SNVs in plants with SpCas9, rBE46b, and rBE49b**. d** Number of nucleotide A/T loci with more than 10 reads to be qualified for calculating the frequency of A>G SNVs in line 49bAG_s2 and four individual T_1_ plants. **e** Number of SNVs and A>G SNVs, and the percentage of A>G SNVs detected in genome data are shown separately for plants with RNA mutations or plants without RNA mutations. (ns) denotes *p*-value > 0.1 (one-tailed Wilcoxon test).


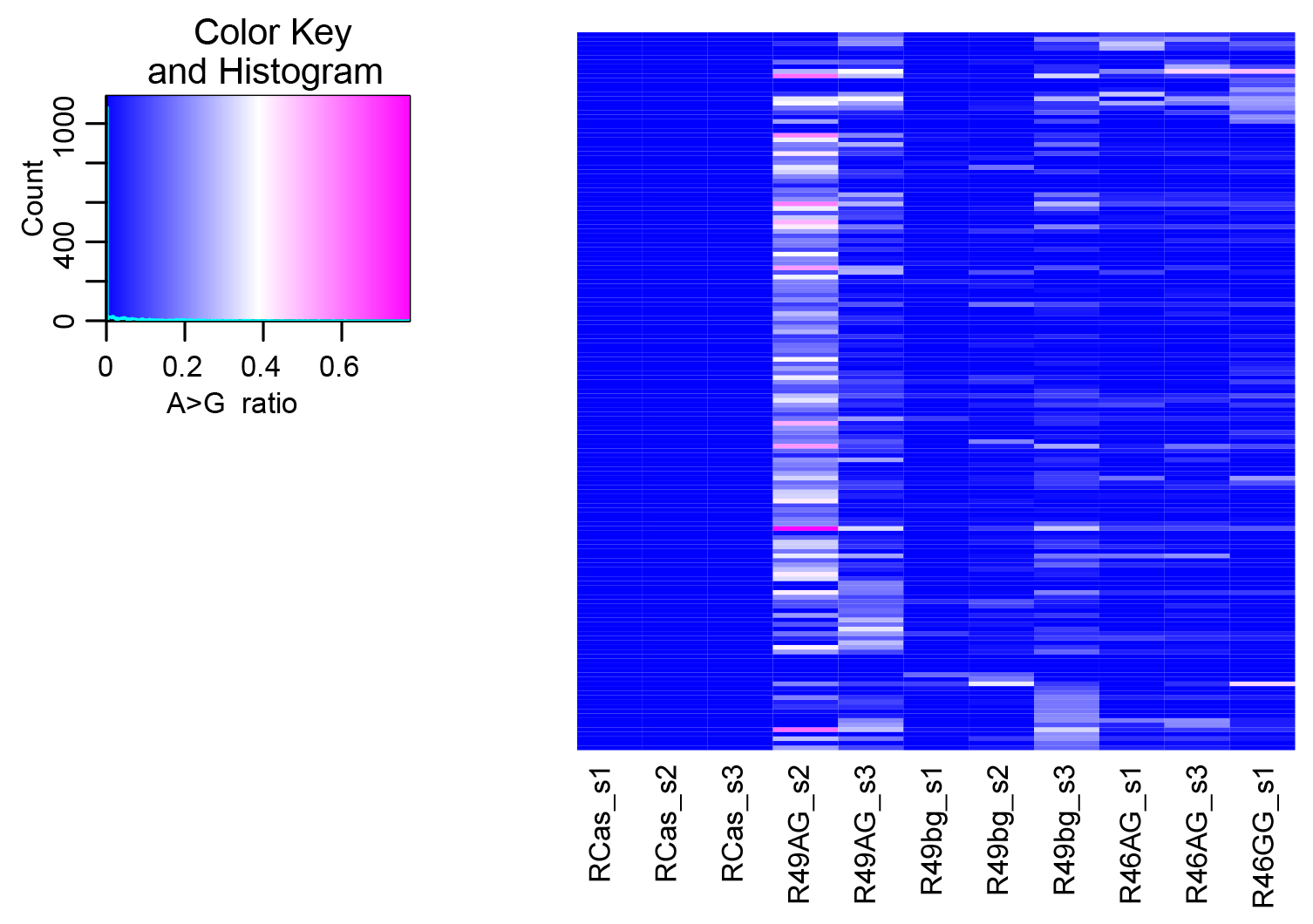


**Figure S14. Heatmap demonstrating A>G mutations in transcriptomes with more than 5 A>G SNVs detected.**

**
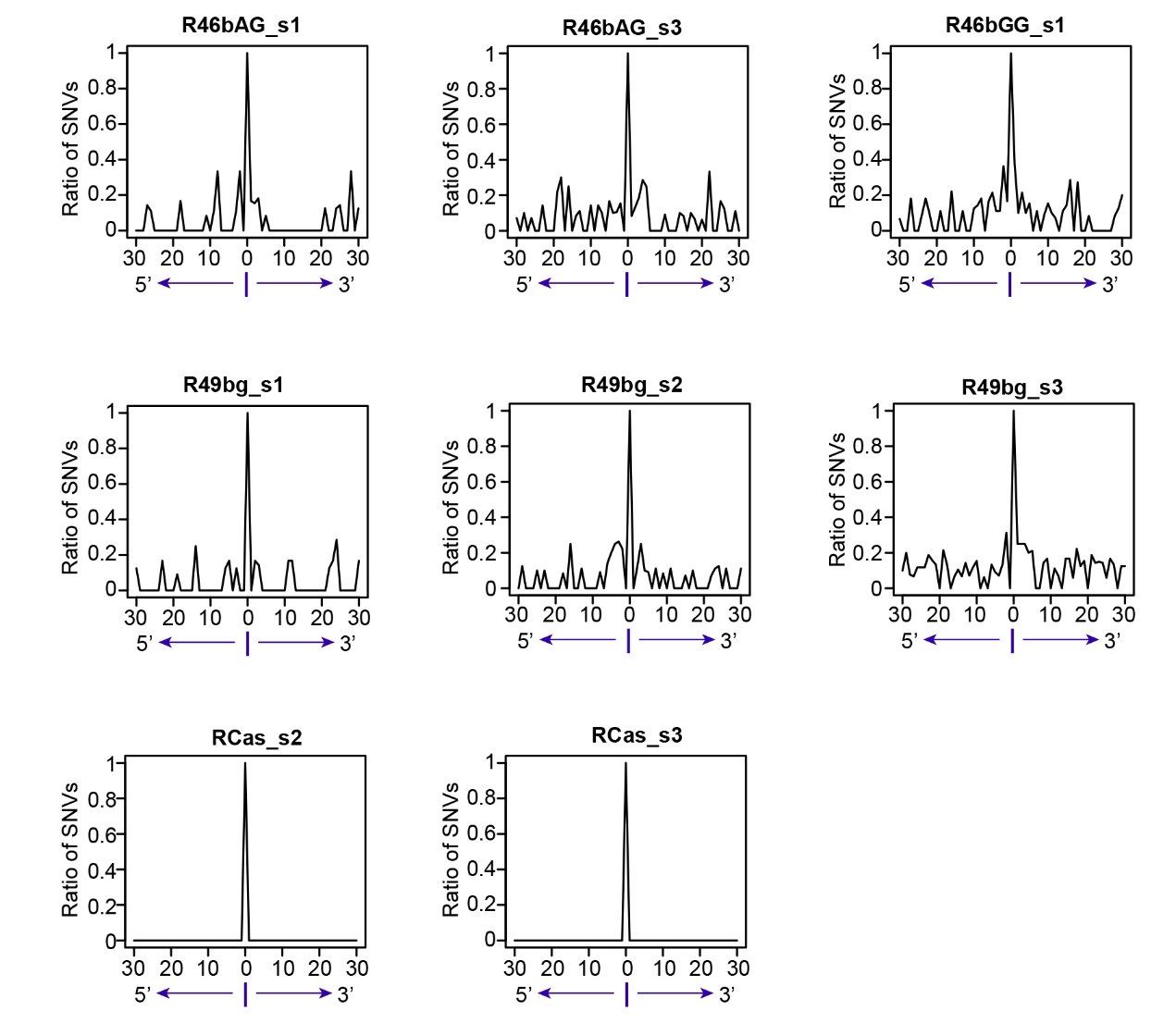
**

**Figure S15. The 5′ and 3′ flanking A>G mutations in transcriptomes with ABEs containing A>G RNA SNVs and in transcriptomes with SpCas9 only lacking A>G RNA SNVs.**

**
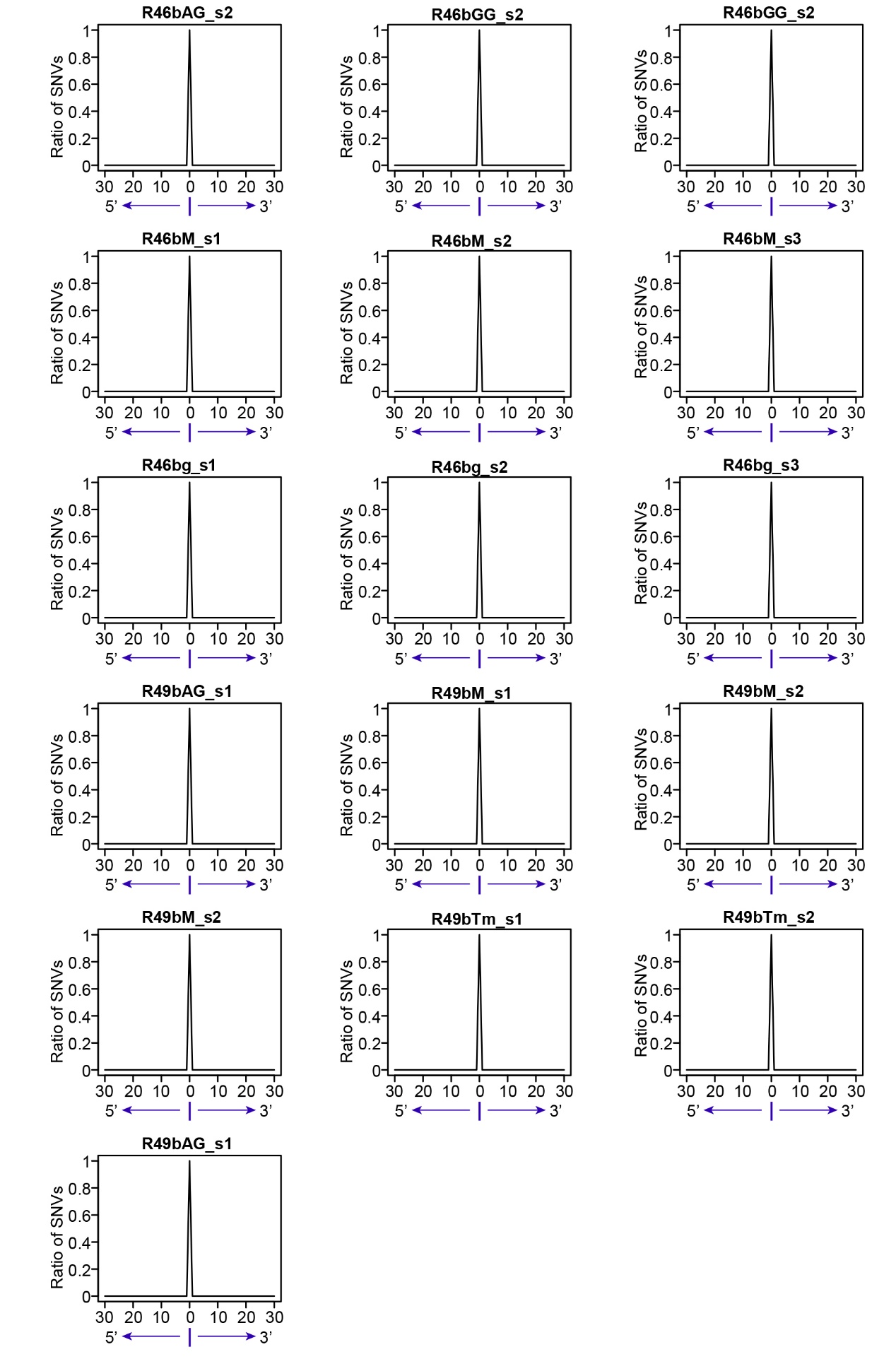
**

**Figure S16. The 5′ and 3′ flanking A>G mutations in transcriptomes with ABEs but without A>G RNA SNVs.**

**
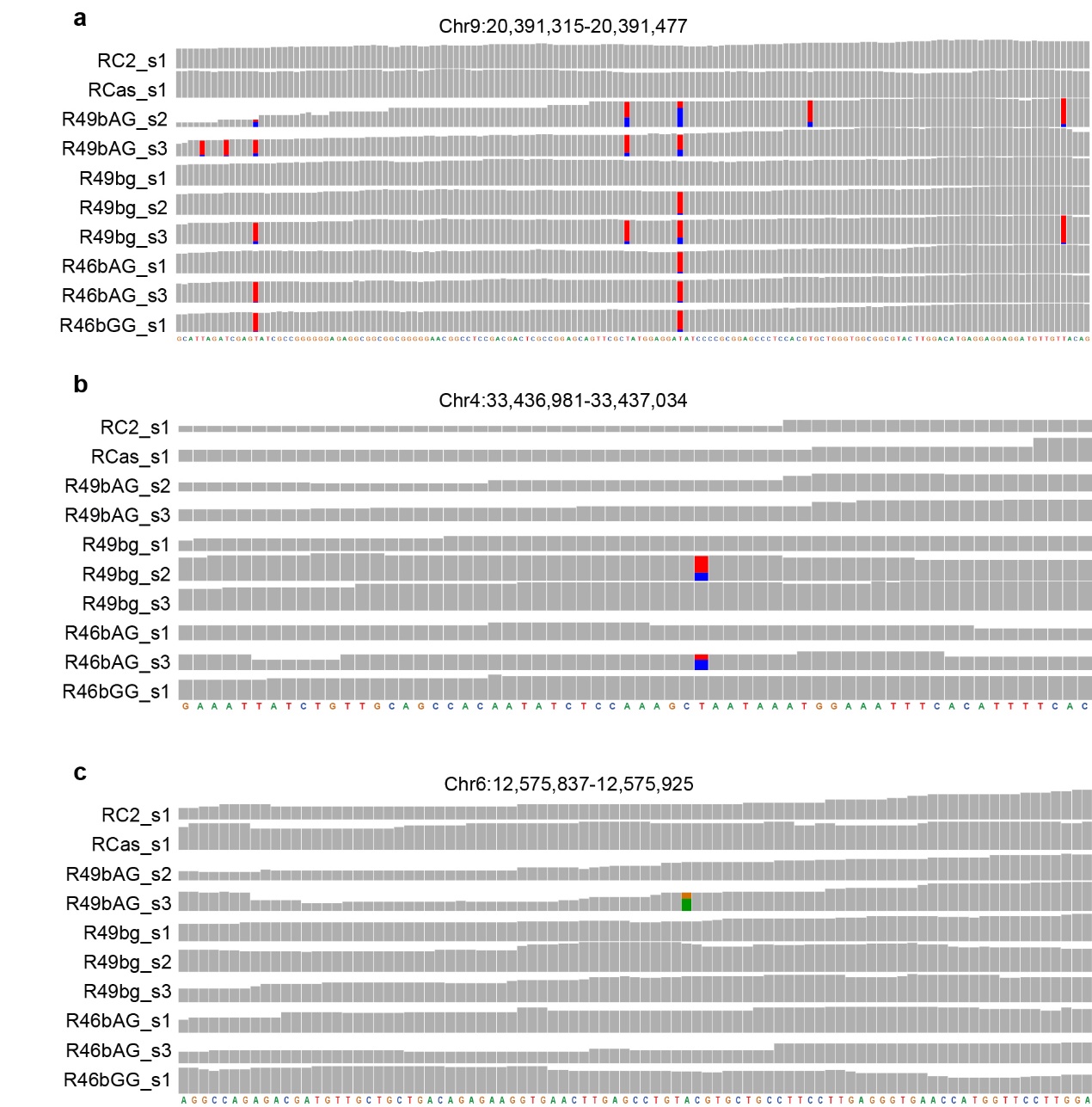
**

**Figure S17. IGV genome browser views showing the off-target RNA mutations. a** IGV genome browser views showing representative loci with clustered A>G SNVs in the transcriptome. **b-c** IGV genome browser views showing representative loci without clustered A>G SNVs in the transcriptome. In IGV genome browser views, the grey bar represents a sequenced nucleotide that is the same as the reference genome, while bars in other colors represent sequenced nucleotides that are partially or totally different from the reference genome: red represents nucleotide T, green represents nucleotide A, orange represents nucleotide G, and blue represents nucleotide C. The height of each color bar represents the relative composition of each nucleotide.

**
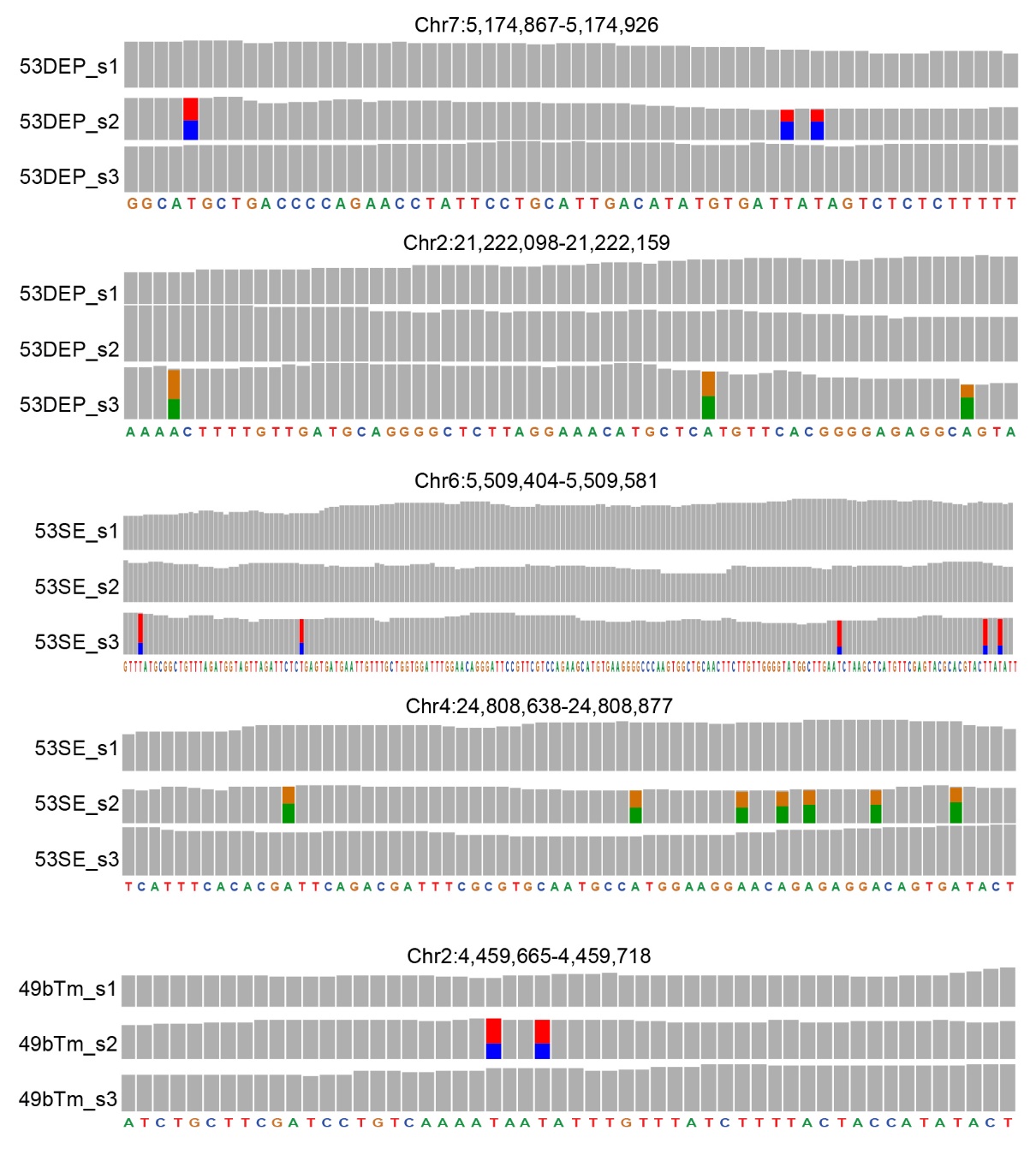
**

**Figure S18. IGV genome browser views showing A>G mutations with flanking A>G SNVs in genome sequencing data.** In IGV genome browser views, the grey bar represents a sequenced nucleotide that is the same as the reference genome, while bars in other colors represent sequenced nucleotides that are partially or totally different from the reference genome: red represents nucleotide T, green represents nucleotide A, orange represents nucleotide G, and blue represents nucleotide C. The height of each color bar represents the relative composition of each nucleotide.


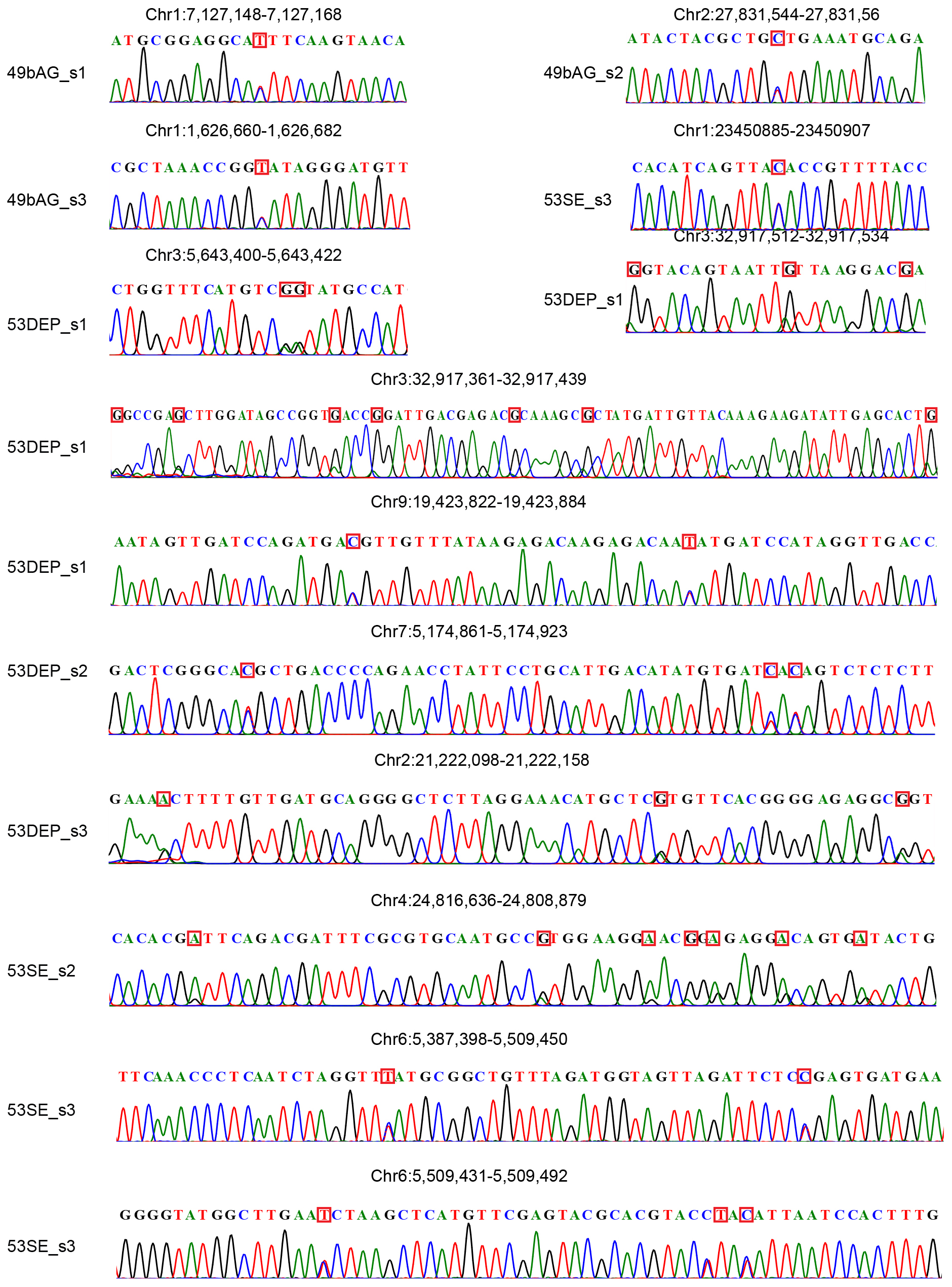


**Figure S19. Sanger sequencing chromatograms of off-target A>G DNA mutations.**


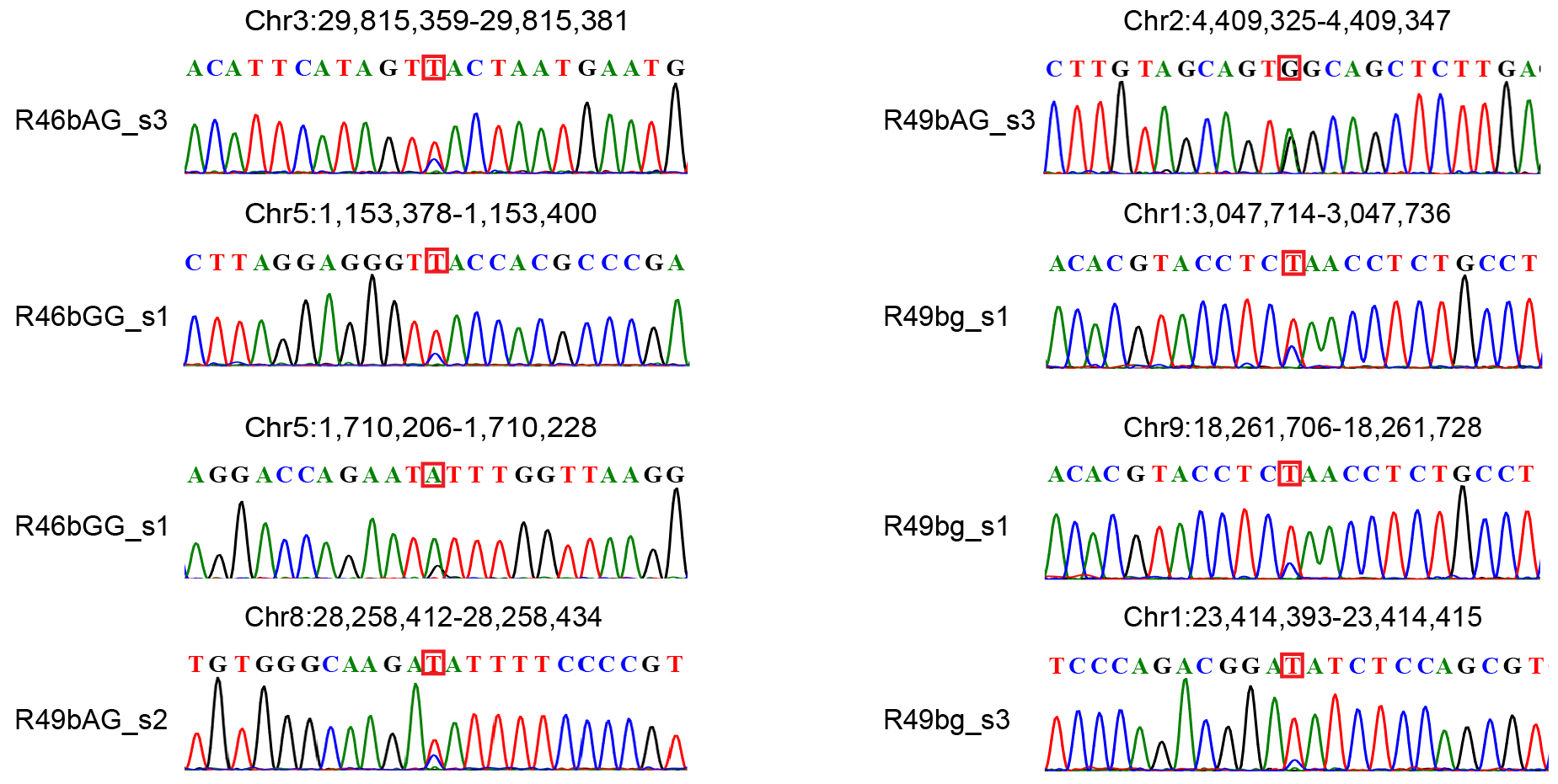


**Figure S20. Sanger sequencing chromatograms of off-target A>G RNA mutations.**
